# Enhancing stratification for survival analyses across standardized data sources

**DOI:** 10.1186/s12911-026-03666-z

**Published:** 2026-07-13

**Authors:** Mikhail Shubov, Mareile Beernink, Jasmin Carus, Alexander Johannes Wiederhold, the AI-CARE Consortium, Christopher Gundler

**Affiliations:** 1 Institute for Applied Medical Informatics, University Medical Center Hamburg-Eppendorf, Martinistr. 52, 20246 Hamburg, Germany; 2 University Cancer Center Hamburg, University Medical Center Hamburg- Eppendorf, Martinistr. 52, 20246 Hamburg, Germany

**Keywords:** Patient stratification, Deep patient representation learning, Clustering, Survival analysis, Data silos, OMOP Common Data Model, Electronic health record, Lung cancer, Interoperability, FAIR principle

## Abstract

**Background:**

Patient stratification is crucial for advancing personalized medicine yet is complicated due to the fragmented and variable nature of healthcare data. The Observational Medical Outcomes Partnership (OMOP) Common Data Model (CDM) addresses the challenges regarding the data by offering a standardized framework for data integration across diverse sources and configurations. Recent advancements in machine learning, particularly transformer-based models such as Bidirectional Encoder Representations from Transformers (BERT), have demonstrated significant potential in extracting deep patient representations from electronic health records.

**Methods:**

This study assesses the efficacy of BERT-based patient representation learning using OMOP CDM data for patient stratification. We harmonize originally incompatible datasets, including the established MIMIC-IV-2.2 and lung cancer data from the German cancer registry Schleswig-Holstein, within the OMOP CDM framework. BERT is pre-trained on the MIMIC-IV-2.2 dataset, and the derived representations are utilized to generate patient embeddings from the cancer registry (test) data. We employ k-means clustering on the embeddings to stratify patient subgroups. To evaluate whether the embeddings are useful for clustering, we divide the original cancer registry data into the corresponding groups and conduct survival analyses on selected columns for each cluster. The clustering method’s effectiveness is assessed by comparing survival models trained on these clusters with naïve clusters derived only from the original dataset. In addition, we included a clinical expert review, in which a physician assessed the resulting cluster assignments for clinical plausibility and interpretability.

**Results:**

Our approach effectively identifies patient similarities across datasets and allowed for efficient patient stratification. Survival analyses show varied performance depending on the model and cluster characteristics, with up to a 11% improvement over naïve k-means clusters, demonstrating the benefits of transfer learning. For some groups of patients, the corresponding accuracy of the survival analysis increased by up to 7%, emphasizing the value of stratifying homogeneous subgroups.

**Conclusion:**

Utilizing standardized data and transformer-based foundation models to generate patient embeddings demonstrates effective knowledge transfer between two vastly different datasets and enables the identification of groups in which the accuracy of survival analysis can be significantly increased.

**Supplementary Information:**

The online version contains supplementary material available at 10.1186/s12911-026-03666-z.

## Background

Patient stratification based on similarities is becoming increasingly important in modern medicine [1, 2]. This approach aligns with the trend towards personalized medicine, which aims to improve therapy and predictions by considering individual differences and medical backgrounds [3]. However, despite the sheer amount of healthcare data available, challenges arise due to the variability, completeness, and interoperability of this data, making it difficult to establish groupings based on a comprehensive dataset [4, 5].

Healthcare data is distributed across numerous data owners (e.g., hospitals, registries, insurance companies, and research centers), with each entity storing data in its own format. This fragmentation of healthcare data is hindering research and collaboration [6]. This missing syntactic interoperability is only a part of the problem. Associated differences in semantic, i.e. due to different recording setups or considered usages, induce further challenges [7]. To address these issues, efforts have been made to unify data silos through common data models (CDMs) and standardization. An example of such is the Observational Medical Outcomes Partnership (OMOP) CDM [8].

In recent years, numerous studies have employed machine learning to create deep patient representations from electronic health record (EHR) data [9]. Several works applied transformer-based language models, Bidirectional Encoder Representations from Transformers (BERT) in particular, to EHR data, achieving better predictive results on their evaluation tasks [10–12]. Despite these advancements, the outcomes of this research still lack generalizability and broader, more unified evaluation tasks and metrics [13].

Initial research started to investigate possible benefits of utilizing standardized data to mitigate differences between datasets used for training transformer models [14]. To further assess the potential benefits of knowledge transfer between data sources using transformer models, we evaluate the usage of a well-established OMOP CDM with patient representation learning using the BERT model. Our approach involves identifying potential similarities between patients despite highly different recording setups: routine clinical data (MIMIC-IV-2.2) used for training the model and cancer registry data from Schleswig-Holstein (Germany) for evaluation. Routine clinical data is collected across diverse healthcare settings and potentially offers a more comprehensive view of patient trajectories [15]. In contrast, cancer registries provide detailed disease-specific information that is collected specifically for research purposes. If we could nevertheless demonstrate the ability of the models to derive and re-use implicit knowledge, this would provide evidence that sharing knowledge across data silos is achievable with limited effort.

To assess the usefulness of the obtained representation (encoding the assumed knowledge) within a realistic task, we employ various models within the domain of survival analysis. Specifically, survival analyses are conducted on subgroups derived from the learned embeddings, where the analysis itself is performed on the original structured data. In a medical context, survival analyses are commonly used to predict the probability of an event occurring over time, often dealing with censored data, where the event of interest may not have occurred for all subjects within the study period. Our evaluation method involves comparing survival models trained on the clustered data with those trained on the entire dataset, using the recent work of Germer et al. [16], as they compared various classical and novel survival models on the same cancer registry data in its entirety.

Related work in the context of survival analysis on stratified population have shown only limited improvements. When performed within individual clusters, the approaches yield results that are almost identical to those obtained from analyzing the entire dataset. For instance, regressions on survival times for lung cancer patients [17] and classifications concerning key outcomes, such as the 5-year survival rate for breast cancer patients [18], have demonstrated only marginal differences when conducted within clusters versus the entire dataset. Additionally, these analyses commonly did not use classical approaches for survival analysis but rephrased the task as challenges without censored data commonly found in epidemiological studies. Therefore, we investigate possible options to improve the previously only mediocre results and make the resulting models more applicable in reality. For that, we assess whether patient representations learned from routine clinical data can be transferred to cancer registry data in a way that allows the resulting clusters to be effective for survival analysis using well-established methods on censored data.

## Methods

**Fig. 1:**
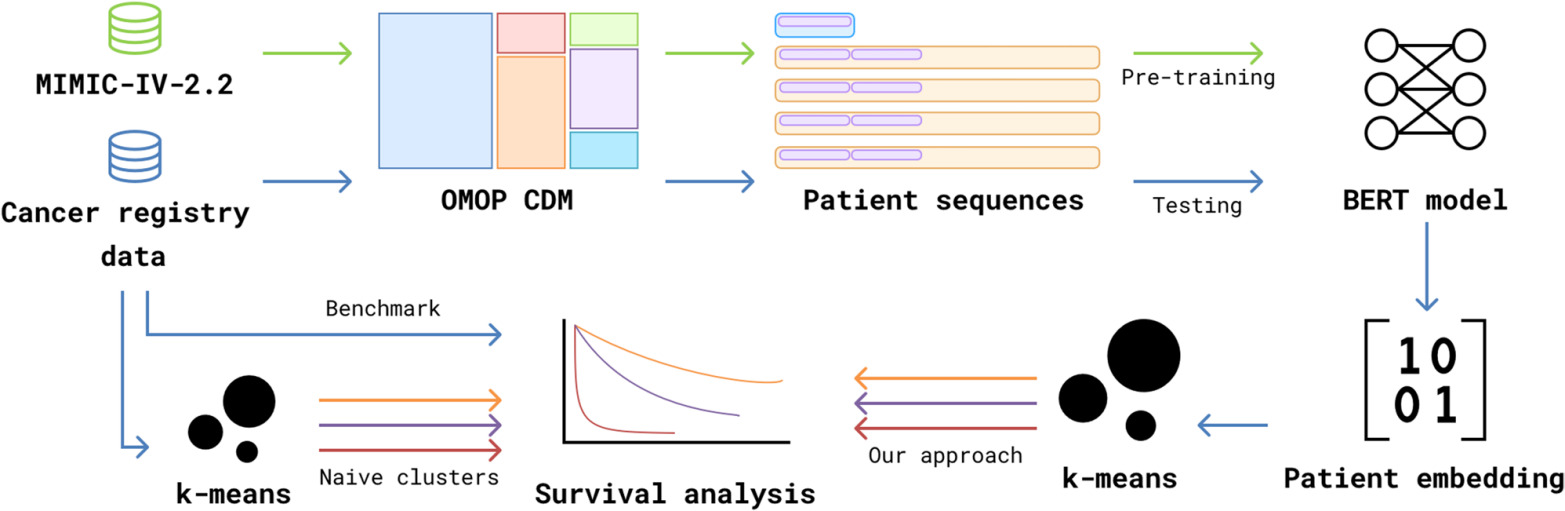
Overview of the process for generating and analyzing patient embeddings. It begins with mapping from original datasets into OMOP CDM, creating a unified patient sequence, pre-training BERT on MIMIC-IV-2.2, and subsequently generating patient embeddings based on the cancer registry data. Following this, the k-means algorithm is applied to create patient subgroups, which are then evaluated using various survival models. While the survival analysis itself is conducted using selected structured features the grouping of patients is of central importance, as it is based on our embedding-driven stratification. The effectiveness of our approach is assessed by comparing survival outcomes from these subgroups to those derived from survival analyses on the entire dataset (benchmark) and clusters formed from the original dataset without embeddings (naïve clusters)

This section presents the methodology for generating patient embeddings from datasets mapped to the OMOP CDM using the BERT model. It also describes how these embeddings are analyzed through clustering and provides an overview of the survival models used for evaluation. Figure 1 provides an overview of the process employed in this paper.

### Data

For assessing the ability to learn across domains, we utilized two distinct yet prototypical datasets. For the clinical example, the MIMIC dataset is a comprehensive, de-identified database containing health-related data from patients who stayed in critical care units of the Beth Israel Deaconess Medical Center in the USA between 2008 and 2019. MIMIC-IV-2.2, the latest iteration released in 2023, includes over 431,231 hospital admissions and 73,181 ICU admissions [19]. The data is organized into three modules: hospital admissions, intensive care unit admissions, and clinical notes, encompassing patient demographics, lab tests, medications, treatments, and observations. The MIMIC data is used for pre-training. As an example of registry data, a dataset from the cancer registry of the federal state Schleswig-Holstein, Germany is used, from which 10,383 lung cancer patients with diagnoses between 2018 and 2022 are utilized [20, 21]. Germer et al. [16] utilized the same dataset in their analysis of state-of-the-art algorithms, making it an attractive benchmark for comparison. The cancer registry data is event-based and not structured around full EHR visits. It contains key oncological events (diagnosis, therapies, medications, death) in separate tables, each linked by patient ID. We mapped these to OMOP CDM using both automated and manual vocabulary mappings. As MIMIC data is also not available in OMOP, we mapped it to this standard. Details regarding the mapping of both datasets and the necessary quality checks could be found in the Appendix A1.

### Model

For generating the interoperable embeddings, we utilized the original BERT architecture [22] with a self-developed tokenization customized for OMOP data. The approach was inspired by the previously published work and enriched with custom improvements: BEHRT [11], a BERT adaptation for EHR data, uses positional embeddings for encoding separate visits, age of the patients and considering the specific information included in the patient sequence, this method only includes diagnostic medical codes. Our work attempts to include dates as input tokens to introduce temporal aspects and includes the following OMOP CDM domains such as *Procedures*, *Observations*, *Drugs*, and *Conditions* to provide more context to the model. G-BERT [12] introduced ontology embeddings for encoding medical concept codes with the hierarchical structure of the medical vocabularies. However, it takes an average of all the patient visits and does not include temporal information. In our work, in contrast, all the patient visits are combined in one sequence to provide the model with the context of patient trajectory and make use of the attention mechanism. The information from the hierarchical structure of medical vocabularies is not included in the encoding process. CEHR-BERT [10], while having a similar approach, did not include the *Observations* domain of the clinical data and used artificial time tokens to mark the time gaps between events. Our work, in contrast, makes the date tokens relative to the birth date of the patients. Accordingly, this approach offers a more natural (age-based) integration of temporal information.

**Fig. 2:**
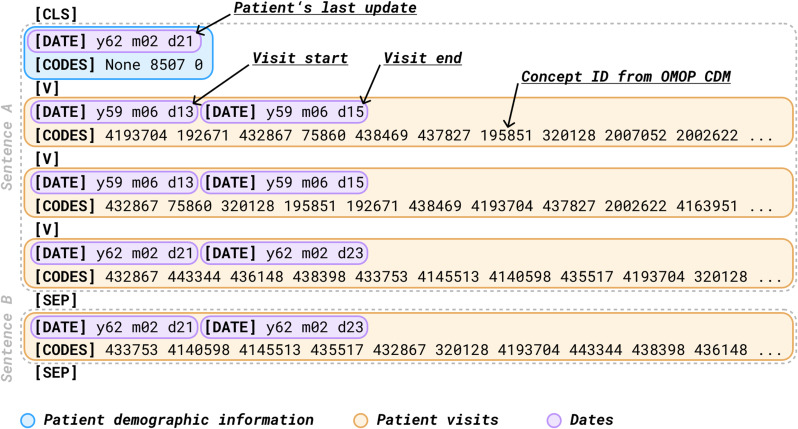
Patient sequence for BERT’s Next Sentence Prediction (NSP) and Masked Language Modelling (MLM) tasks. Special tokens: [CLS] - BERT’s classification token; [DATE] – date encoded with year, month and day tokens; [CODES] – marks the beginning of the section, where visit-related medical data is presented by Concept IDs from OMOP CDM; [V]– separates patient visits; [SEP] - separates sentences A and B the end of the sequence

Patient data from OMOP CDM was transformed into sequences suitable for the model (Fig. 2). Each sequence, representing one patient, included demographic data followed by visit-specific medical codes, separated by special tokens. The medical codes are the Concept IDs (from the following OMOP CDM domains: *Condition Occurrence*,* Drug Exposure*,* Observation*,* Procedure Occurrence*) associated with each visit. Dates were converted to year, month, and day tokens relative to the patient’s birth date.

To create patient representations, both the tokenizer and the model were trained on the OMOP-mapped MIMIC-IV-2.2 data while holding the cancer registry data out as a fully separate test set. Therefore, not all the tokens within the latter dataset’s sequences were encoded using the vocabulary for the tokenizer fixed at training time. Nevertheless, 84% of tokens in the cancer registry data sequences were successfully encoded. The BERT model itself was pre-trained using the Masked Language Modelling (MLM) objective and the Next Sentence Prediction (NSP). For the latter objective, for a patient with *N* visits *N-1* positive pairs are generated, where *sentence A* includes demographic information and visits from 1 to k-1 and *sentence B* includes visit k (k: from 2 to N). Similarly, *N-1* negative pairs were generated by taking a random visit from another patient in the dataset (ensuring the date is after the last visit of sentence A to prevent the model from learning the “shortcut”). The model was trained for 1 M steps, with a batch size of 8 and linear learning rate scheduling with warmup ratio of 0.1. The latest model and the tokenizer are available on the Hugging Face Hub [23]. The embeddings including the knowledge learned by the model were acquired from the CLS token (BERT’s classification token) of the last hidden state of the BERT model. These embeddings, of size 768, were then generated for the cancer registry data to enable the evaluation regarding their usefulness.

## Evaluation

### Selecting and understanding clusters

Given the high dimension of the embeddings, dimensionality reduction techniques (PCA, UMAP, t-SNE) were used to visualize the structure of the embeddings. PCA explained variance showed the number and contribution of components. The patient embeddings were categorized into clusters by the k-means algorithm with the Elbow Method to select the optimal number of clusters. We calculated gender distribution, average visit count, and token count in the sequence as basic characteristics per cluster.

To gain further insight into the resulting clusters, the most common tokens within each cluster were analyzed by calculating percentage of patient sequences having each unique token and sorting them by their odds ratio (OR). Details on the OR calculation method are provided in the Appendix A2. The tokens were converted to their textual description (*Concept Name*) that is stored within the standardized vocabularies of OMOP accessible through the platform Athena. This allows us to inspect the most common tokens in each cluster and observe the meaningful grouping of the patients. Additionally, a clinical expert review was conducted, in which a physician evaluated the cluster assignments for clinical plausibility and interpretability.

### Survival analysis

To evaluate whether the stratification is suitable for survival analyses, we selected Cox proportional hazards model (CoxPH), Random Survival Forest (RSF), and DeepSurv with TabNet architecture as survival analysis models. In general, the choice of models and metrics was informed by the comparative analysis conducted by Germer et al. [16] utilizing the same dataset, where our selection of features is based on their proposed TNM (tumor, node, metastasis) subset. Accordingly, the survival analysis focuses on evaluating the impact of key features, including sex, age at diagnosis, histology group and the TNM staging parameters regarding tumor size, the number of involved lymph nodes, and the presence of metastasis. While the survival models are trained using these clinical features, the grouping of patients on which the analyses are conducted is derived from our prior stratification based on learned embeddings.

CoxPH [24] is a widely used linear model in survival analysis that estimates the effect of each variable on survival, producing a vector of coefficients corresponding to the logarithm of hazard ratio. Using the fitted model, a patient-specific survival function can be predicted, which indicates the probability of surviving beyond a given time point. RSF [25] drops each data sample down the trees in the forest until reaching terminal nodes, where the data is used to estimate survival and cumulative hazard functions non-parametrically. The ensemble prediction is the average across all trees in the forest. DeepSurv [26], utilizing the TabNet architecture, is a deep neural network designed to model interactions between a patient’s variables to predict individual risk. It outputs a single node estimating the risk function, with network parameters trained using a negative logarithmic partial likelihood loss function derived from CoxPH. Instead of the original DeepSurv architecture, we choose the TabNet architecture [27] for its demonstrated effectiveness in classifying and regressing tabular data, supported by initial evaluations indicating it may offer improved results [16].

The predictive performance of the models is evaluated using several well-established and comparable metrics. The concordance index (C-I) indicates the fraction of all comparable pairs where the model correctly predicts a higher risk for the sample with a shorter survival time than another sample with a longer survival time. The integrated Brier score (IBS) measures the mean squared error between the predicted probabilities of remaining event-free (no death or 0) up to a specified time point and the actual vitality status. It calculates this metric across different time intervals, evaluating specifically at each 10% quantile of observed events. Additionally, the mean area under the curve (AUC) is computed as a cumulative measure of the receiver operating characteristic curve, indicating the models’ ability to distinguish between subjects at different risk levels over time. The mean AUC scores are also averaged across each 10% quantile of events.

Missing values are addressed using two distinct approaches: first, by treating missing values as a separate category, and second, by estimating them using the Miss Forest imputation technique. For categorical columns, both label encoding and one-hot encoding are applied. To enhance training stability, we employ a 5-fold cross-validation strategy during the model development process.

## Results

In the results section we analyze the properties of the clusters and evaluate their usefulness for survival analysis. Given its extensive nature, our evaluation of the mapping quality of MIMIC-IV-2.2 and cancer registry data to OMOP CDM is available in the Appendix A1.

### Evaluation of embeddings

Reflecting the complexity of datasets, dimensionality reduction techniques (PCA, UMAP, t-SNE) revealed no distinct clusters or structure. 19 PCA components captured 90% of variance in the cancer registry data. The Elbow Method indicated an optimal cluster number of six for the cancer registry data. However, based on the results of the experiments regarding the TabNet survival model, additional cluster sizes were investigated for the survival analyses. Therefore, the results for TabNet with a cluster number of three are presented and discussed in detail as a complementary analysis.

Several features were calculated for each cluster of cancer registry data (Table 1). Cluster 3 has longer sequences (mean 194.3 tokens) and more patient visits (mean 12.1 visits). On the other hand, cluster 1 has shorter sequences (mean 26.8 tokens) and fewer patient visits (mean 1.3 visits) – with many patients having one visit. Cluster 0 has balanced genders and an average number of visits (mean 4.9 visits), with an average sequence length (mean 90.5 tokens). Similarly, cluster 5 has a visit count of 5.2 visits and sequence length of 84.6 tokens. Clusters 2 and 4 also display average visit counts and sequence lengths, with cluster 4 having a higher mean number of tokens (59) and a higher visit count (mean 3.8 visits) than cluster 2.

**Table 1:** Cancer registry data: cluster analysis

Cluster		Gender		Visit Count		# tokens	
	Total (n)	Male (%)	Female (%)	Mean	Median	Mean	Median
0	1275	572 (44.86)	703 (55.14)	4.9	5	90.5	88
1	1687	668 (39.60)	1019 (60.40)	1.3	1	26.8	23
2	1726	705 (40.85)	1021 (59.15)	2.7	2.5	45.7	43
3	1753	745 (42.50)	1008 (57.50)	12.1	11	194.3	177
4	1997	820 (41.06)	1177 (58.94)	3.8	3	59.0	53
5	3203	1311 (40.93)	1892 (59.07)	5.2	5	84.6	81
Overall	11,641	4821 (41.41)	6820 (58.59)	5.0	4	83.3	64

The OR metric was used to find the top concepts for each cluster of both datasets. Based on these top concepts, an overview of differences and overlaps between the clusters can be assessed. In cancer registry data, each cluster represents a different spectrum of oncological patient trajectories, with cluster 0 and 3 standing out as the most distinct based on the average OR values of all the tokens within one cluster. Cluster 0 has an average OR of 1.52 (22% than the mean of all clusters) and cluster 3 has 2.25 (80% higher than the mean). The clusters included patients with concepts related to palliative care and end-of-life treatments, featuring older patients based on their time tokens and those undergoing longer and more complex oncological treatments. Appendix A2 provides an overview of the defining characteristics of each individual cluster. The full tables of most common tokens are located in the GitHub repository [28].

The embedding-derived clusters were clinically reviewed by a physician using the cluster-specific distributions of age, sex, histology group, TNM categories, UICC stage, sequence characteristics, and enriched OMOP concepts. From a clinical perspective, the clusters were medically plausible and reflected meaningful differences in disease stage, treatment trajectory, and documentation density. Cluster 0 was dominated by diagnostic and pathological staging concepts and appeared to represent a more clearly staged subgroup with lower metastatic burden. In contrast, clusters 3 to 5 corresponded more closely to advanced disease trajectories, with cluster 3 characterized by chemotherapy- and immunotherapy-related concepts and prolonged treatment courses, cluster 4 by radiotherapy-related concepts, and cluster 5 by palliative care patterns. Cluster 1 was associated with older age, sparse trajectories, and end-of-life related concepts, whereas cluster 2 showed a more surgery- and intensive-care-oriented profile. Overall, these findings support the clinical relevance of the learned embeddings, although the identified clusters should be interpreted as composite clinical phenotypes rather than definitive biological subtypes.

### Survival analysis on stratified clusters

To assess the suitability of the stratified clusters for survival analysis, we first perform the analyses within each cluster, using the defined clinical features. Then, we compare the results with those from the entire dataset, which serves as our benchmark and was reported by Germer et al. [16].

Table 2 presents the survival analysis results for the stratified clusters, using a default cluster size of 6. The aggregated results, which represent the mean values across all clusters, indicate that the CoxPH and RSF models achieve comparable performance across all metrics, while TabNet underperforms relative to these models. Slightly better than the RSF model, the CoxPH model with one-hot encoding nearly always achieves the highest performance. All models show improved performance under the Miss Forest imputation technique, with the advanced techniques (Miss Forest imputation and one-hot encoding) particularly influencing the performance of the CoxPH model.

**Table 2:** Aggregated survival analysis results on clusters using k-means with default k = 6 on embeddings

Classifier	C-I	IBS	Mean AUC
**No Imputation**			
CoxPH	0.646 ± 0.004	0.170 ± 0.005	0.700 ± 0.014
CoxPH*	**0.681 ± 0.006**	**0.155 ± 0.007**	**0.743 ± 0.009**
RSF	0.674 ± 0.005	0.158 ± 0.008	0.732 ± 0.010
RSF*	0.677 ± 0.007	0.158 ± 0.008	0.737 ± 0.009
TabNet	0.591 ± 0.008	0.247 ± 0.015	0.634 ± 0.009
TabNet^§^	0.651 ± 0.009	0.179 ± 0.011	0.700 ± 0.008
**Miss Forest Imputation**			
CoxPH	0.693 ± 0.007	**0.151 ± 0.005**	**0.757 ± 0.007**
CoxPH*	0.693 ± 0.009	**0.151 ± 0.006**	**0.757 ± 0.008**
RSF	0.691 ± 0.007	0.153 ± 0.005	0.752 ± 0.006
RSF*	**0.694 ± 0.009**	**0.151 ± 0.003**	**0.757 ± 0.010**
TabNet	0.604 ± 0.012	0.258 ± 0.013	0.627 ± 0.022
TabNet^§^	0.668 ± 0.007	0.220 ± 0.037	0.724 ± 0.013

C-I: concordance index; IBS: integrated Brier score; AUC: area under the curve

* results where one-hot encoding is used instead of label encoding; ^§^ values for a cluster size of 3; Bold: best values

When comparing different metrics, where higher scores on the C-I and mean AUC, and lower scores on the IBS metric indicate better performance, all models achieve higher performance on the mean AUC compared to the C-I metric. The choice of cluster size has minimal impact on CoxPH and RSF, although TabNet’s performance improves with fewer clusters. Thus, the performance gap between the models decreases with a smaller number of clusters, although TabNet still falls of the performance of the CoxPH and RSF models.

**Fig. 3:**
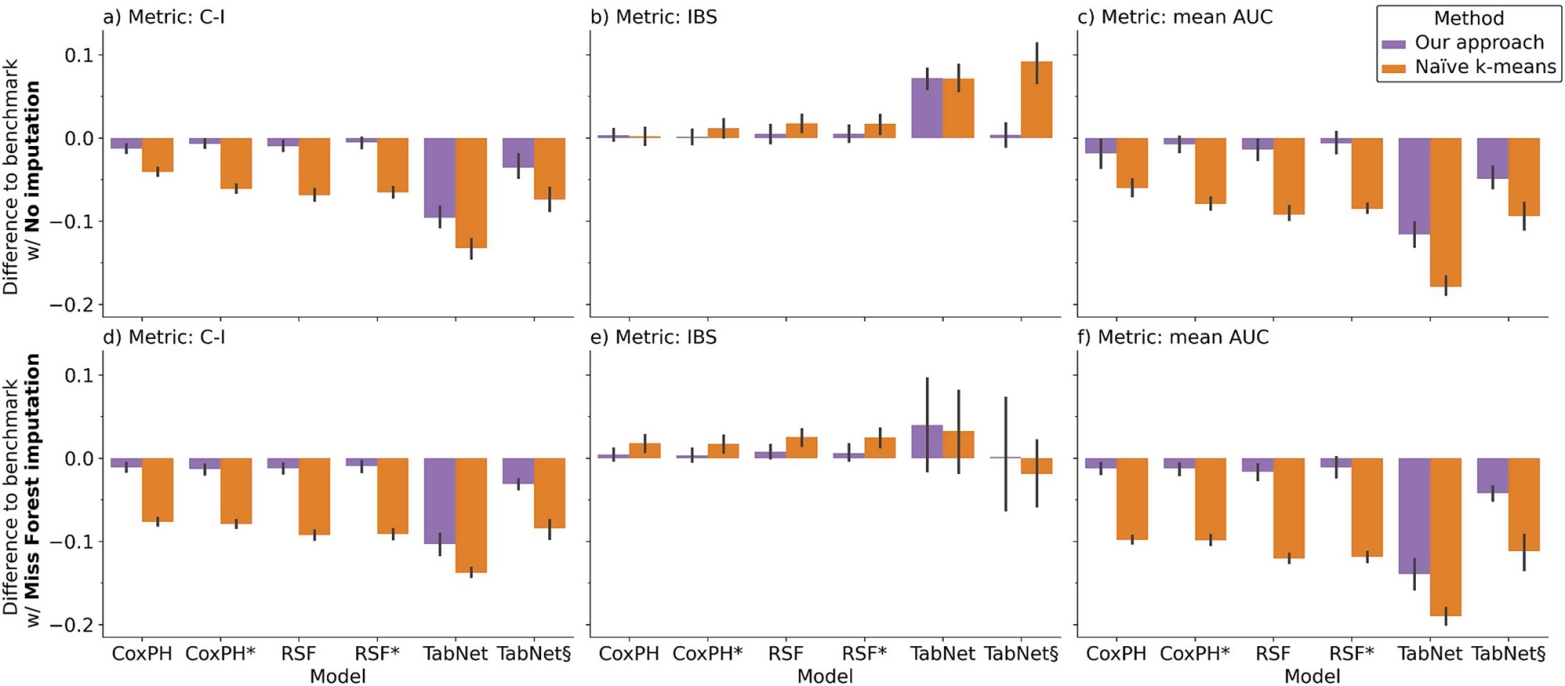
Differences in survival analysis results, where the benchmark (reported in [16]) has been subtracted from the aggregated results of two types of clusters: one created from embeddings (purple) and the other from the original dataset (orange). Both types of clusters use k-means with the same default number of clusters, k = 6. Plots **a-c** (top) show results for no imputation, while **d-f** (bottom) show results for Miss Forest imputation. For all metrics, a value of 0 indicates the same performance as the benchmark. For concordance index (C-I) (**a, d**) and mean area under the curve (AUC) (**c, f**), more negative values indicate worse performance, while larger positive values in the integrated Brier score (IBS) (**b, e**) signify worse performance compared to the benchmark. * results where one-hot encoding is used instead of label encoding; § values for a cluster size of 3

Comparing these results with the benchmark shows an overall decline in performance, though the decrease is minimal for the CoxPH and RSF models, with a maximum difference of 2% and common decline of 1%. However, TabNet experiences significant performance drops, up to 14% with six clusters. This difference can be mitigated by using fewer clusters (Fig. 3).

Additionally, we quantify the benefits of the learning across datasets by ruling out a possible influence of the clustering procedure itself. We created the clusters not only from the embeddings (our approach) but also compared them to k-means on the features directly selected from the original dataset. Compared to the benchmark, the survival rates for these naïve clusters show significantly larger performance differences than those observed with our approach. In particular, metrics such as the C-I and mean AUC, as well as the use of the Miss Forest imputation technique show significant deterioration. For the classifiers CoxPH and RSF, the decline ranges from 4% to 12%, while TabNet experiences a decline of up to 19%. Figure 3 illustrates the aggregated scores for our approach and the naïve k-means clusters in comparison to the benchmark. Apart from the IBS results for the TabNet model, our approach consistently outperforms the naïve method, while also demonstrating comparable performance to the benchmark when using CoxPH and RSF models.

The analysis of individual clusters based on embeddings reveals performance variations across clusters, as shown for C-I metric in Table 3. With a cluster size of six, the benchmark can be exceeded by up to 7%, except for the TabNet model. While some clusters (especially cluster 0) demonstrate enhanced performance, others, such as cluster 3, fall below the benchmark, a trend consistently observed across all three metrics. As previously noted, the performance of the TabNet model improves with fewer clusters; however, it remains below the benchmark results for all clusters. Further analyses indicate that excluding certain clusters like cluster 0 (representing a small portion of the data), has minimal impact on overall performance, as detailed in Table 3.

**Table 3:** Survival analysis results of the concordance index (C-I) metric for each individual cluster compared to the benchmark (reported in [16]). In addition to the results of each cluster, the analysis outcome for a combined dataset (clusters 1 to 5) are presented

Classifier	Cluster 0	Cluster 1	Cluster 2	Cluster 3	Cluster 4	Cluster 5	Cluster 1 - Cluster 5
**No Imputation**							
CoxPH	*0.07 ± 0.03*	-0.01 ± 0.01	**-0.04 ± 0.00**	**-0.08 ± 0.01**	-0.01 ± 0.01	0.01 ± 0.01	-0.01 ± 0.00
CoxPH*	*0.06 ± 0.02*	-0.01 ± 0.01	-0.02 ± 0.01	**-0.08 ± 0.01**	-0.01 ± 0.01	0.00 ± 0.01	-0.01 ± 0.00
RSF	*0.06 ± 0.02*	-0.01 ± 0.01	-0.02 ± 0.00	**-0.08 ± 0.01**	-0.01 ± 0.00	0.00 ± 0.01	-0.01 ± 0.00
RSF*	*0.06 ± 0.02*	0.00 ± 0.01	-0.02 ± 0.01	**-0.07 ± 0.02**	-0.01 ± 0.01	0.00 ± 0.00	0.00 ± 0.00
TabNet	**-0.13 ± 0.03**	**-0.05 ± 0.01**	**-0.10 ± 0.04**	**-0.19 ± 0.00**	**-0.09 ± 0.03**	-0.02 ± 0.03	-0.01 ± 0.00
TabNet^§^	-0.02 ± 0.03	**-0.04 ± 0.01**	**-0.05 ± 0.02**	-	-	-	-
**Miss Forest Imputation**							
CoxPH	*0.05 ± 0.02*	*0.03 ± 0.02*	-0.02 ± 0.01	**-0.09 ± 0.01**	**-0.03 ± 0.01**	0.00 ± 0.01	-0.01 ± 0.00
CoxPH*	*0.05 ± 0.02*	0.02 ± 0.01	-0.02 ± 0.01	**-0.08 ± 0.01**	-0.02 ± 0.01	0.00 ± 0.01	-0.01 ± 0.00
RSF	*0.05 ± 0.02*	0.01 ± 0.03	-0.02 ± 0.01	**-0.09 ± 0.01**	**-0.03 ± 0.01**	-0.01 ± 0.01	-0.01 ± 0.00
RSF*	*0.05 ± 0.02*	*0.03 ± 0.01*	-0.01 ± 0.01	**-0.09 ± 0.01**	**-0.03 ± 0.01**	0.00 ± 0.00	-0.01 ± 0.00
TabNet	**-0.13 ± 0.03**	**-0.11 ± 0.03**	**-0.10 ± 0.03**	**-0.11 ± 0.02**	**-0.11 ± 0.04**	**-0.07 ± 0.02**	-0.01 ± 0.00
TabNet^§^	**-0.03 ± 0.02**	-0.01 ± 0.00	**-0.05 ± 0.01**	-	-	-	-

* results where one-hot encoding is used instead of label encoding; ^§^ values for a cluster size of 3; Bold: differences exceeding 3% worse performance; Italics: differences exceeding 3% better performance

## Discussion

In the context of current research in the medical domain, data itself is gaining increasing attention and importance. In complex, heterogeneous, and global fields like medicine, relying solely on evidence from a single data source as the “truth” is particularly risky. Therefore, interoperable data from diverse origins is crucial for creating robust machine learning models. The process of transforming infrastructure and mapping data can be both time-consuming and costly. However, when our aim is to extract meaningful insights from data and apply them in different contexts, it becomes a necessity.

Our study illustrates the advantages of utilizing standardized data across two vastly different data sources and demonstrates how previously unavailable knowledge can significantly enhance performance in survival analysis through the stratification of the patient population.

As a necessary precondition, our findings confirm that modern transformer algorithms can effectively learn from standardized data at the syntactic level. Patient sequences were created using medical codes and dates, employing a tokenization and encoding approach with a fixed vocabulary, which proved effective in encoding patient sequences. Despite training on data from a large hospital in the USA with minimal cancer-related data, the model successfully encoded 84% of tokens in a special dataset from a single German cancer registry focused on secondary cancers, specifically lung cancer. If such vast differences in data collection purposes, locations, health insurance systems, clinical phenotypes, treatment practices, and other factors do not pose a significant challenge, this supports the validity of a generalizable approach applicable to many other standardized data sources.

Learning from large, general datasets to small, specialized setups, as conceptualized in foundation models, holds significant potential in the context of oncological survival analyses. Using naïve clustering with the k-means algorithm as a baseline, we demonstrated that, for that specific algorithm and task, no additional information present in the data from a cancer registry led to effective stratification. In fact, the reduced amount of data available within each cluster for subsequent survival analysis algorithms resulted in significant performance decreases up to 19%. However, once we utilized patient similarities learned from the training data as “additional information” and based clustering on these, we improved predictions for specific patient cohorts by up to 7%. Even if these improvements were smaller for other groups, the additional information generally offset the impact of the reduced sample size for all methods but TabNet. Accordingly, these improvements provide evidence of a much-needed quality in machine learning: learning semantically and generalizing across diverse datasets through standardized data.

These findings suggest that the clusters capture clinically relevant differences in patient trajectories which are not fully represented by conventional variables such as TNM, histology, age, and sex alone. This is important as it indicates that embedding-based stratification may add clinically meaningful structure to survival analyses across standardized data sources, beyond what can be achieved by naïve clustering on the original feature set.

### Limitations and future work

Given the methodological complexity, this study has several limitations that suggest areas for further research.

One approach is to enhance the capabilities of the task-agnostic foundation model. At least three possible strategies exist. First, future work could enhance the training process. The original BERT model benefited from training on approximately 3.3 billion tokens, while the dataset derived from MIMIC-IV-2.2 used here contains fewer than 169 million tokens. Given the nature of standardized data, future studies could incorporate other datasets, expanding the training data’s volume and variety. It may also be beneficial to extend the BERT model’s pre-training duration and employ a more advanced learning rate scheduling scheme. Secondly, increasing the model’s input size could allow the processing of longer patient sequences. This could be achieved using a larger BERT variant or a different architecture designed for handling longer inputs. As advancements in large language models continue, new pre-training methods and architectural innovations are introduced that could be utilized for further research, potentially replacing BERT. Third, patient sequence encoding could benefit from the integration of external knowledge, such as the hierarchical relationships between medical concepts in standardized medical vocabularies. These enhancements could lead to a more powerful foundation model based on the OMOP CDM.

While the OMOP CDM ensures data harmonization, we acknowledge that the clinical complexity of patient data may lead to embeddings that don’t fit well into spherical clusters, as assumed by methods like k-means. Future work may benefit from alternative clustering approaches that better reflect the structure of complex patient data. Although the improved survival prediction metrics in several clusters suggest that the embeddings capture clinical information, the chosen approach to clustering (k-means and Elbow method) does not guarantee direct alignment with clinically relevant phenotypes. To address this, the resulting clusters were additionally reviewed by a physician. This clinical assessment supported the validity of the identified clusters and confirmed their relevance from a medical perspective. To improve the specific task of stratifying patients for survival analysis, several strategies remain available. Incorporating more similar cases specifically related to the cancer entity of interest is a straightforward strategy but requires retraining. Alternatively, it could be beneficial to use features other than those selected by Germer et al. [16]. Additionally, utilizing supervised projection algorithms for feature selection could introduce additional knowledge without altering the foundation model. Finally, we would like to clarify that the evaluation via survival analysis is just one of several possible evaluation methods. The review by Wornow et al. [13] demonstrates that foundation models can be effectively tested and applied to a range of medical questions, offering opportunities for further exploration in future research.

## Conclusions

In this study, we explored the potential of leveraging standardized data across vastly differing data sources to enhance survival analysis in medical research. Our findings underscore the opportunities of utilizing those to develop robust and generalizable machine learning models for estimating similarities between patient trajectories. The resulting patient stratifications on German cancer registry data resulted in significantly improved survival analyses for some groups of patients.

The evidence presented further highlights the necessity and advantage of transforming traditional data infrastructures to enable interoperability and facilitate comprehensive data integration. While these transformations can be resource-intensive, the ability to derive actionable insights for example by automatically matching patients to applicable clinical trials and generalize findings across various contexts appears to justify the effort. Overall, the study reaffirms the value of standardized data in medical informatics, setting the stage for further exploration and refinement of machine learning techniques capable of bridging the gap between heterogeneous datasets. As we continue to develop these models, harnessing their full potential will require ongoing collaboration and innovation within the medical and data science communities.

## Supplementary Information

Below is the link to the electronic supplementary material.

Supplementary Material 1

## Data Availability

The cancer registry data is available on reasonable request and after the corresponding clearance process from the cancer registry Schleswig-Holstein, Germany. All reported values are publicly available in the Appendix, GitHub repository [28] and Hugging Face [23].
