# Supplementary material for: Enhancing stratification for survival analyses across standardized data sources
Source: BMC Med Inform Decis Mak. 2026 Jul 13;26:261. doi: 10.1186/s12911-026-03666-z (PMC13361719; doi:10.1186/s12911-026-03666-z)
Supplement: Supplementary file 1 — Supplementary Material 1 [file 12911_2026_3666_MOESM1_ESM.docx]

# Enhancing Stratification for Survival Analyses across Standardized Data Sources

[Mikhail Shubov](https://orcid.org/0009-0006-3678-0290), [Mareile Beernink](https://orcid.org/0009-0001-2793-196X), Jasmin Carus, Alexander Johannes Wiederhold, the AI-CARE Consortium and Christopher Gundler

**The AI-CARE Consortium includes:**

Alice Nennecke^2^, Henrik Kusche^2^, Vera Heinrichs^2^, Andrea Eberle^3^, Sabine Luttmann^3^, Khalid Abnaof^4^, Soo-Zin Kim-Wanner^4^, Bernd Holleczek^5^, Katharina Rausch^5^, Natalie Rath^5^, Heinz Handels^6^, Sebastian Germer^6^, Marco Halber^7^, Martin Richter^7^, Martin Pinnau^8^, David Reinert^8^, Jannik Schaaf^8^, Holger Storf^8^, Tobias Hartz^9^, Nils Goeken^9^, Janina Bösche^9^, Alexandra Stein^10^, Kerstin Weitmann^10^, Wolfgang Hoffmann^10^, Louisa Labohm^11^, Alexander Katalinic^11,12^, Christiane Rudolph^12^, Christopher Gundler^1^, Mareile Beernink^1^, Frank Ückert^1^

**Author details:**

^1^ University Medical Center Hamburg-Eppendorf, Institute for Applied Medical Informatics, Martinistraße 52, 20246 Hamburg, Germany. ^2^ Hamburg Cancer Registry, Ministry of Science, Research, Equality and Districts, Free and Hanseatic City of Hamburg, Süderstraße 30, 20097 Hamburg, Germany. ^3^ Bremen Cancer Registry, Leibniz Institute for Prevention Research and Epidemiology - BIPS, Achterstraße 30, 28359 Bremen, Germany. ^4^ Hessian Cancer Registry, Hessian Office of Health and Care, Lurgiallee 10, 60439 Frankfurt, Germany. ^5^ Saarland Cancer Registry, State Ministry of Labour, Social Affairs, Women and Health, Neugeländstraße 9, 66117 Saarbrücken, Germany. ^6^ German Research Center for Artificial Intelligence (DFKI), Ratzeburger Allee 160, 23562 Lübeck, Germany. ^7^ Baden-Wuerttemberg Cancer Registry, Klinische Landesregisterstelle Baden-Württemberg GmbH, Birkenwaldstraße 149, 70191 Stuttgart, Germany. ^8^ Johann Wolfgang Goethe-Universität Frankfurt, Universitätsklinikum Frankfurt, Institut für Medizininformatik, Theodor-Stern-Kai 7, 60590 Frankfurt am Main, Germany. ^9^ Clinical Cancer Registry Lower Saxony, Sutelstraße 2, 30659 Hannover, Germany. ^10^ Institute for Community Medicine, Section Epidemiology of Health Care and Community Health, University Medicine Greifswald, Ellernholzstraße 1-2, 17475 Greifswald, Germany. ^11^ Institut für Sozialmedizin und Epidemiologie, Universität zu Lübeck, Ratzeburger Allee 160, 23538 Lübeck, Germany. ^12^ Institut für Krebsepidemiologie an der Universität zu Lübeck, Registerstelle des Krebsregisters Schleswig-Holstein, Ratzeburger Allee 160, 23562 Lübeck, Germany

## A1. Mapping of data and associated quality checks

To ensure syntactic and semantic interoperability, both datasets were mapped to the OMOP CDM v5.3 through an Extract-Transform-Load (ETL) process. For the cancer registry data of Schleswig-Holstein, a custom ETL process was designed to map the dataset to OMOP CDM. This process involves:

1. Linking source data fields with the OMOP CDM columns using Rabbit In a Hat^[[1]](#footnote-2)^ and ATHENA^[[2]](#footnote-3)^ tools to create an overview of the mapping.
2. Correcting data format errors and standardizing NULL values.
3. Executing the ETL process in SQL or Python, ensuring data is uploaded to a relational database.
4. Using tools like the Data Quality Dashboard (DQD)^[[3]](#footnote-4)^ to evaluate the mapping quality.

The cancer registry data ETL process, designed from scratch, required iterative refinement and collaboration with domain experts. Key challenges included determining appropriate mappings for source data values to the OMOP CDM structure and addressing custom German medical vocabularies. The ETL process treated separate source codes (e.g. diagnoses, drugs, therapies) from the source data tables as distinct entities and mapped them to the corresponding standard concepts in OMOP. The Table A1.1 shows the overview of the source data tables, their description and to which OMOP tables the data was mapped. The following vocabularies were used during the ETL process: SNOMED, Gender, Cancer Modifier, OMOP Genomic, OMOP Extension, NAACCR, ICDO3, ICD10GM, ICD10PCS, RxNorm.

Table A1.1: Cancer registry data: ETL process overview, linking source data tables to the OMOP CDM tables.

| **Table** | **Description** | **OMOP CDM Tables Targeted** |
| --- | --- | --- |
| Tumoren.csv | Primary tumor registry | Condition_occurrence  Observation  Measurement  Person  Death |
| SY.csv | Systemic therapies | Procedure_occurrence  Observation  Drug_exposure |
| ST.csv | Radiotherapy | Procedure_occurrence  Observation |
| OP.csv | Surgeries | Procedure_occurrence  Observation  Condition_occurrence |
| MET_PT.csv,  MET_Verlauf.csv | Metastasis types and timeline | Measurement |
| VM.csv | Vital status | Death |

All patients were successfully mapped to OMOP using relations between concepts from ATHENA. Approximately 80 source code values required manual mapping and expert confirmation to ensure accuracy. The DQD evaluation for cancer registry data (Table A1.2) showed a 99% pass rate (97% for applicable checks). More than 3000 checks are not applicable in the case of cancer registry data due to the small size of the dataset and the fact that it does not use all the OMOP CDM columns.

Table A1.2: Cancer registry data DQD Evaluation Results


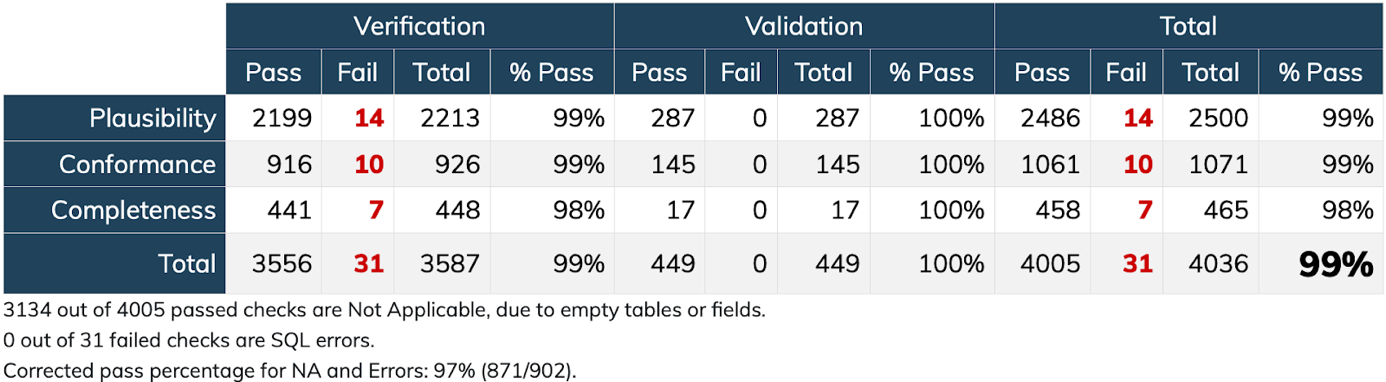


An open-source ETL script^[[4]](#footnote-5)^ from the OHDSI community is used to map MIMIC-IV-2.2 data to OMOP CDM, but it involved several adjustments and modifications to the original OHDISI scripts. Key modifications included:

- Correcting the formatting issues that caused DATETIME stamps to be treated as strings,
- Excluding the Waveform data module due to its limited patient coverage (only 100 patients),
- Fixed birthdate calculation with anchor_year and anchor_age,
- Modified the script to function correctly on UNIX-based systems,
- For specific tables (hosp_emar_detail, hosp_icd_diagnoses, and hosp_pharmacy), we conducted thorough data cleaning and uniform handling of missing values.

After executing the ETL process, the data was evaluated using the DQD. The results indicated a 92% pass rate for the checks, rising to 94% when considering only applicable checks, which is sufficient for the use-case of the data.

Table A1.3: MIMIC-IV-2.2 DQD Evaluation Results


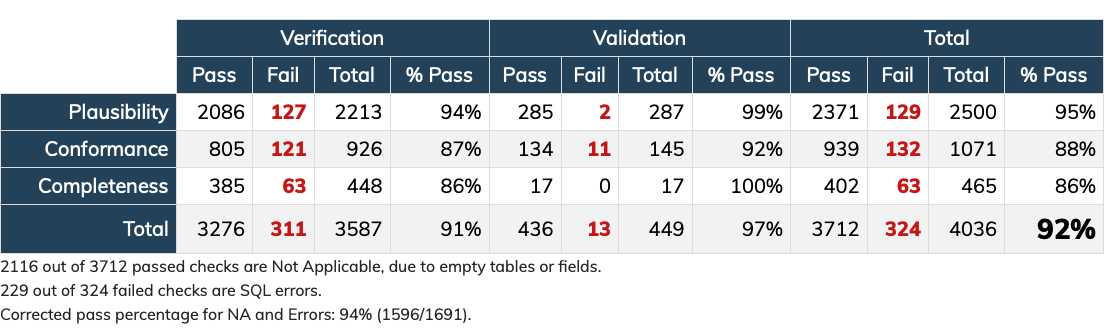


## A2. Cancer registry data: Analysis of most common tokens within clusters.

To find out how the clusters are different from one another and the dataset on average, two approaches were tried:

**Percentage Ratio (PR):** This metric is calculated by first determining the percentage of sequences within a particular cluster that contain a particular token ***t***, and then calculating the percentage of sequences in the entire dataset that contain that token. The ratio of these two percentages is then used to compare the prevalence of the token within the cluster to the whole dataset. Let ***n_c,t_*** be the number of sequences containing token *t* in cluster ***c***, and ***N_c_*** be the total number of sequences in cluster ***c***. Let ***n_t_*** be the number of sequences containing token ***t*** in the whole dataset, and ***N*** be the total number of sequences in the dataset. The formula for the percentage ratio *PR_t_* is given by:

Equation A2.1: Percentage Ratio (PR)

$$PR_{t}=\frac{\left( \frac{n_{c,t}}{N_{c}} \right)}{\left( \frac{n_{t}}{N} \right)}$$

**Odds Ratio (OR):** This metric is used to compare the odds of a sequence in a particular cluster containing a particular token ***t*** with the odds of any sequence in the entire dataset containing that token. Using the same notation as above, the odds of having token ***t*** in cluster ***c*** is $\frac{n_{c,t}}{N_{c}-n_{c,t}}$, and the odds of having token ***t*** in the entire dataset is$\frac{n_{t}}{N-n_{t}}$. The odds ratio *OR_t_* is then calculated as:

Equation A2.2: Odds Ratio (OR)

$$OR_{t}=\frac{\left( \frac{n_{c,t}}{N_{c}-n_{c,t}} \right)}{\left( \frac{n_{t}}{N-n_{t}} \right)}$$

The assumption is that the higher PR and OR scores show those concepts that dominate the cluster. However, PR seems to bring to the top rare medical concepts that have low absolute percentages. For example, 0.47% of the patients of the cancer registry embeddings have the concept *Impaired wound healing*, the percentage on the whole data is 6.71 times lower, making it a significant concept for the cluster. However, the other more frequent concepts, such as cT category, show an increase of 2.14 within the cluster while being present in 80.95% of the patient sequences. This makes it a significant concept for the cluster, even though it has a smaller percentage ratio. But OR shows the ability to balance between these two cases: therefore, it was chosen to find the top concepts for each cluster of both datasets. The full list of tokens contained in the clusters can be found in the Excel table (clustering_cancer_registry.xlsx) in the GitHub repository^[[5]](#footnote-6)^.

- **Cluster 0** is predominantly characterized by diagnostic and pathological classifications such as *Diagnostic Confirmation, cT, cN, cM, pT, pN, and pM categories*, and *Adenocarcinoma*. The presence of *Pathological Grades* and specific anatomical descriptions like *Structure of upper lobe* *of lung* indicate a focus on cancer staging and tumor characterization. This cluster includes detailed pathological staging, such as *AJCC/UICC pathological* and *clinical stages*.
- **Cluster 1**, in contrast, is marked by a higher incidence of end-life care and procedures related to severe chronic conditions, as indicated by *Death of unknown cause* and procedures like *Excision of lesion of meninges* and *Microlaryngoscopy*. The inclusion of *year tokens* ranging from 84 to 111 suggests a tendency towards older patients, which aligns with the cluster's focus on terminal medical interventions.
- **Cluster 2** shares some overlap with cluster 1, particularly in the appearance of *Death of unknown cause*. However, it distinguishes itself with a higher frequency of thoracic surgeries (*surgical procedure on thorax*, *thoracoscopic procedure*) and conditions often associated with intensive care scenarios (*intensive care monitoring*, *adenocarcinoma with squamous metaplasia*). This cluster seems particularly engaged with critical surgical procedures.
- **Cluster 3**: treatment-related concepts, esp. *chemotherapy*, *immunotherapy* for cancer, and specific medications such as *durvalumab*, *dexamethasone*, and *carboplatin*. This cluster, having the longest patient sequences and the largest number of visits and tokens, may represent a subset of patients undergoing complex cancer therapy (*radiation therapy* and *chemotherapy changed* - *progressive disease during chemotherapy*)
- **Clusters 4** and **5** focus on specialized therapeutic and palliative care approaches. Cluster 4 with emphasis on radiation therapy and specific surgical interventions, and cluster 5 concentrating on palliative measures.

1. GitHub - OHDSI/WhiteRabbit: WhiteRabbit is a small application that can be used to analyse the structure and contents of a database as preparation for designing an ETL. It comes with RabbitInAHat, an application for interactive design of an ETL to the OMOP Common Data Model with the help of the scan report generated by White Rabbit. <https://github.com/OHDSI/WhiteRabbit>, [accessed August 18, 2024] [↑](#footnote-ref-2)
2. Athena. <https://athena.ohdsi.org/>, [accessed April 17, 2024] [↑](#footnote-ref-3)
3. Blacketer C, Schuemie FJ, Ryan PB, Rijnbeek P. Increasing trust in real-world evidence through evaluation of observational data quality. J Am Med Inform Assoc. 2021;28:2251–7. doi: 10.1093/jamia/ocab132 [↑](#footnote-ref-4)
4. GitHub - OHDSI/MIMIC: MIMIC (Medical Information Mart for Intensive Care) is a large, single-center database comprising information relating to patients admitted to critical care units at a large tertiary care hospital. This repository contains the ETL to the OMOP CDM. --- github.com. <https://github.com/OHDSI/MIMIC>, [accessed August 18, 2024] [↑](#footnote-ref-5)
5. UKE IAM. (2024). AI-CARE-Consortium/omop-stratification. <https://github.com/AI-CARE-Consortium/omop-stratification> [accessed October 9, 2024] [↑](#footnote-ref-6)
